# Dosing patterns of canakinumab in patients with Cryopyrin-Associated Periodic Syndromes (CAPS): A comparative analysis of a study in Western versus Japanese patients

**DOI:** 10.1186/1546-0096-9-S1-P12

**Published:** 2011-09-14

**Authors:** B Bader-Meunier, P Hachulla, J Kuemmerle-Deschner, M Gattorno, N Patel, R Preiss, K Lheritier, T Imagawa, R Nishikomori, H Takada, T Heike, T Hara, S Yokota

**Affiliations:** 1 Department of Pediatric Immunology and Rheumatology, Université Paris-Descartes and Hôpital Necker-Enfants Malades, Assistance Publique Hôpitaux de Paris, Paris, France; 2 On behalf of the Canakinumab D2306 and D2308 study group

## Background

CAPS is an orphan auto-inflammatory disease, generally diagnosed in childhood that requires life-long treatment. Canakinumab, a fully human anti-IL-1b antibody, has previously demonstrated rapid, complete and sustained response in CAPS patients.

## Aim

To compare dosing patterns of canakinumab in pediatric and adult CAPS patients of a predominantly Western population (WP) vs Japanese patients (JP).

## Methods

**Figure 1:**
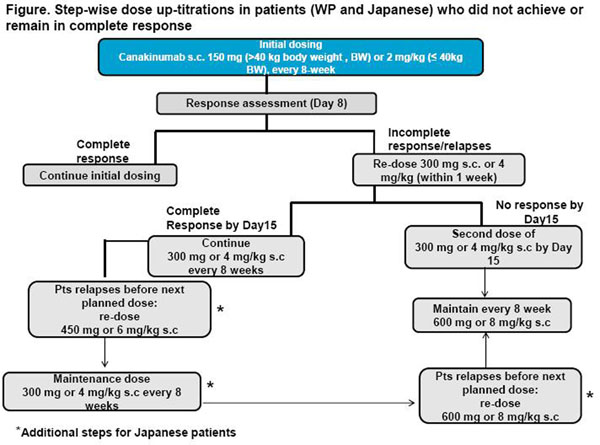
Step-wise up-titrations in patients (WP and Japanese) who did not achieve or remain in complete response

Canakinumab s.c. 150 mg (if >40 kg) or 2 mg/kg (if ≤40 kg) was dosed every 8 weeks. Step-wise up-titrations in dose were allowed in patients who did not achieve/remain in complete response (CR, Figure 1).

## Results

Median duration of treatment was 414 (29-687) days in WP and 337 days (59-373 days) in JP. In the WP, CR was achieved in 85/109 (78%) canakinumab-naive patients. 127/141 (90%) evaluable patients remained in CR throughout the study. 47/166 patients in WP and 11/19 patients in the Japanese study were pediatrics. 36.2% vs 81.8% (WP vs JP) of children received up-titrated and/or more frequent doses. Higher median doses were required in pediatric patients in the JP compared with WP to control MWS and NOMID (Table 1). 13% vs 45% (WP vs JP) of the children received the maximum permitted dose. None of those children showed an unusual type or frequency of adverse events.

**Table 1:** Canakinumab doses by phenotypes

Phenotype (n=WP/JP)	Wester population		Japanese population	
	Adult^1^ Mean/median (mg) (N=136)	Pediatrics^2^ Mean/mdeian (mg/kg) (N=29)	Adult^1^ Mean/median (mg) (N=8)	Pediatrics^2^ Mean/mdeian (mg/kg) (N=11)

MWS (103/7)	200/150	5.5/4.0	225/150	6.0/6.0
NOMID (32/11)	299/150	5.8/4.0	300/225	5.5/6.0
FCAS (30/0)	189/150	2.7/2.0	-	-

## Conclusions

Increased doses of canakinumab were equally efficacious in patients of a WP and Japanese population comprising different CAPS phenotypes without evidence of a change in AE profile. These data suggest that children and patients with more severe CAPS phenotypes, irrespective of ethnicity, require differential dosing.

